# Femtosecond-Laser-Ablation Dynamics in Silicon Revealed by Transient Reflectivity Change

**DOI:** 10.3390/mi13010014

**Published:** 2021-12-23

**Authors:** Tao Feng, Gong Chen, Hainian Han, Jie Qiao

**Affiliations:** 1 Chester F. Carlson Center for Imaging Science, Rochester Institute of Technology, 54 Lomb Memorial Drive, Rochester, NY 14623, USA; afeng304362@163.com (T.F.); gc6200@rit.edu (G.C.); hnhan@iphy.ac.cn (H.H.); 2 Shanghai Institute of Optics and Fine Mechanics, Chinese Academy of Sciences, 390 Qinghe Road, Shanghai 201800, China

**Keywords:** femtosecond laser ablation, silicon laser ablation, two-temperature model, Drude model, two ablation regimes

## Abstract

The dynamics of ablation in monocrystalline silicon, from electron-hole plasma generation to material expansion, upon irradiation by a single femtosecond laser pulse (1030 nm, 300 fs pulse duration) at a wide range of fluences is investigated using a time-resolved microscopy technique. The reflectivity evolution obtained from dynamic images in combination with a theoretical Drude model and a Two-Temperature model provides new insights on material excitation and ablation process. For all fluences, the reflectivity increased to a temporary stable state after hundreds of femtoseconds. This behavior was predicted using a temperature-dependent refractive index in the Drude model. The increase in velocity of plasma generation with increasing fluence was theoretically predicted by the Two-Temperature model. Two ablation regimes at high fluences (>0.86 J/cm^2^) were identified through the measured transient reflectivity and ablation crater profile. The simulation shows that the fluence triggering the second ablation regime produces a boiling temperature (silicon, 2628 K).

## 1. Introduction

Femtosecond (fs) laser pulses with short duration and high peak intensity [1] are widely applied to the areas of micromachining [2,3], medical surgery [4,5], and biological analysis [6]. Their significant advantage resides in material removal (or ablation) on the micro/nanometer-scale through nonlinear optical absorption, as well as the separation of energy deposition and material ablation in the time domain that enables “cold” ablation. In the past decades, the fundamental interaction of femtosecond laser pulses with silicon (Si) has been studied extensively [7,8,9,10,11,12,13,14,15] due to its tremendous technological importance, such as in integrated circuits manufacturing.

Optical pump-probe techniques with ultra-short laser pulses have been widely used for studying the dynamic process of ablation in both temporal and spatial domains during the interaction of laser radiation with semiconductor materials [8]. A series of images capturing surface evolution are obtained by imaging the region excited by a pump pulse and illuminated by a probe pulse at different relative delays. Downer et al. first used this technique to analyze the ablation of bulk Si in 1985 [8]. Von der Linde et al. further investigated the physical mechanism of the ablation process, revealing the generation of high-density carriers [9] and the structure of the ablation layer, which causes temporally evolving Newton rings [10,16]. The high reflectivity that appeared at a delay of less than one picosecond (ps) illustrates a rapid direct transition from the solid to the liquid state (termed as nonthermal melting) [17]. This ultrafast process was verified using X-ray diffraction [18,19,20]. Solis et al. studied the electron-hole (e–h) plasma generation, relaxation, phase transition, and the structuring of different materials by measuring the reflectivity change using the dynamic images [21,22,23,24,25,26]. These analyses revealed the relaxation times of the free-electron plasma and the relationship between the crater depth and laser-beam fluence. Two types of imaging geometries have been used for pump-probe imaging. The front-view imaging geometry was mainly used to obtain reflectance and structuring information, with the angle between pump and probe pulses ranging from 0° to ~70° [8,9,10,16,17,21,22,23,24,25,26,27,28]. The side-view imaging geometry (or shadowgraph) was primarily used to investigate laser-induced material ejection and expansion, for which the angle between pump and probe beam is 90° [13,27,29].

The pump-probe-imaging investigations of the ultrashort laser interaction with Si were mainly focused on two scenarios. The first one focused on e–h plasma generation at relatively low fluence (up to the ablation threshold) and within the 1-ps material response time [9]. The second scenario focused on studying the expansion velocity of the ablated materials under relatively high fluence (above 1 J/cm^2^) and at a time delay of tens of picoseconds or even more, mainly through analyzing the growth of Newton rings or shadow-graphic images [13,27]. There has been little investigation on the physical mechanisms of laser–material interaction from 0 to several pico-seconds after irradiation. There has also minimal investigation for fluences reaching several times the ablation threshold.

We present a detailed study of the excitation and ablation processes in monocrystalline silicon using a time-resolved pump-probe system with femtosecond temporal resolution. The transient characteristics after plasma generation and the underlying physical mechanisms are investigated. Section 2 presents the experimental setup. Section 3 analyzes the reflectivity evolution and the associated physical processes at the center of the excited region using the time-resolved images captured at various fluences ranging from 0.5 to 1.75 J/cm^2^.

## 2. Pump-Probe Imaging System

**Figure 1:**
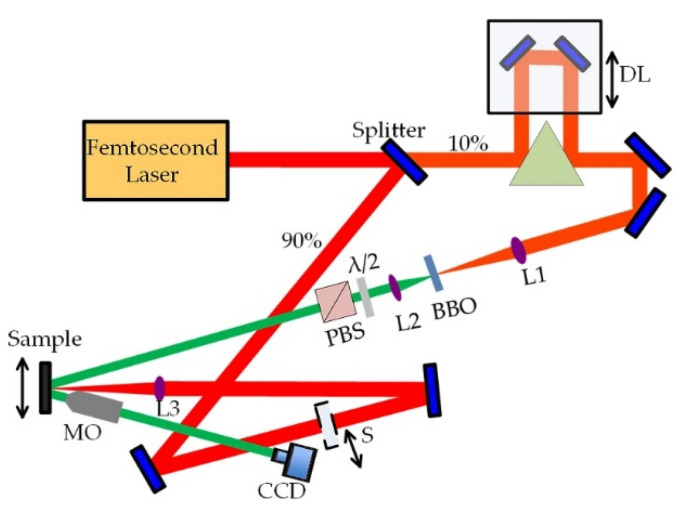
Scheme of the experimental setup. Abbreviations: DL—delay line; L—lens; BBO—nonlinear crystal; λ/2—halfwave plate; PBS—polarized beam splitter; MO—microscope objective; CCD—camera; S—beam shutter.

A schematic diagram of the fs pump-probe imaging setup is shown in Figure 1. A femtosecond laser (Satsuma HP3, Amplitude Laser Group, Pessac, France) was used for all experiments. The laser was operated at a central wavelength of 1030 nm, a temporal width of 300 fs, a repetition rate of 2 MHz, and maximum pulse energy of 40 μJ. The laser pulse was split into the pump and probe paths using a 90/10 beam splitter. The pump path was focused onto the sample surface at normal incidence, with a focal spot diameter of 67 μm (measured at 1/e^2^ peak intensity). The probe beam passed through an optical delay line and was frequency-doubled to 515 nm using a Beta Barium Borate (BBO) crystal. The maximum delay time between the pump pulse and probe pulse was 1.3 ns. The combination of a half-wave plate and a polarizing beam splitter cube controlled the probe beam energy.

The probe pulse illuminates the excited region on the sample at an incidence angle of 31° relative to the sample surface normal. The reflected light was imaged onto a CCD camera (The Imaging Source, DMK 23U274) using a microscope objective (Mitutoyo, M-Plan-NIR, 10×, NA = 0.28).

The zero-delay time of the delay line was defined when the pump and probe pulse were fully overlapped in the temporal domain. It was initially determined by measuring the autocorrelation of the two beams on the BBO crystal and then accurately determined by measuring the reflectivity change induced by laser-excited e–h plasma. The maximum overlap of pump and probe pulse was achieved when the reflectivity change reaches half of the maxima [28].

At each specified laser fluence, the relative reflectivity was calculated using Equation (1) by taking a sequence of three images of the sample surface at various delay times. The first image recorded the original reflectivity *R*_0_ and surface structure, taken 2 s before the arrival of the pump pulse. The second image recorded reflectivity *R_t_* at a specified delay time Δ*t*. The third image recorded the permanent surface change, taken 2 s after the sample was irradiated by the pump pulse. It was noted that the sample was moved to a new position after each shot to ensure that a pristine surface was irradiated by the next pump pulse.

(1) $$\frac{\Delta R}{R}=\frac{R_{t}-R_{0}}{R_{0}}$$

## 3. Results and Analysis

### 3.1. Ablation Dynamics Observation and Analysis

**Figure 2:**
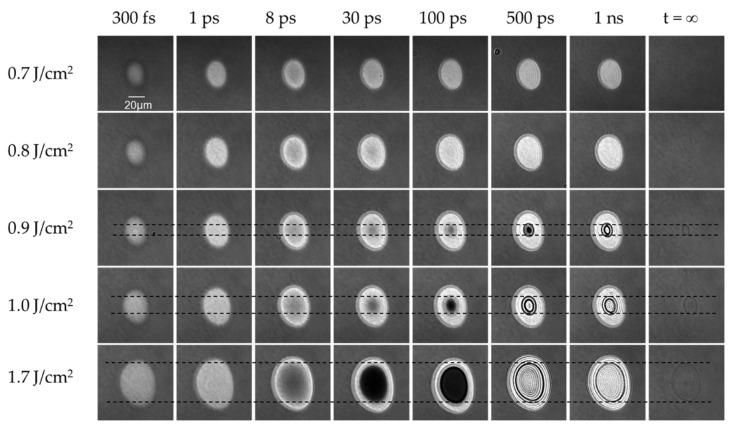
Time-resolved dynamic images captured at different relative pump-probe delays after excitation with a single pulse at different peak fluences. The black dashed horizontal lines indicate the white ring between two black rings (shown at a time delay of 1 ns) due to the permanent change on Si surface.

The impact of laser fluence on ablation dynamics was investigated via comparing the pump-probe images at delay times ranging from 300 fs to 1 ns, using three different fluences. Figure 2 shows the five series of time-resolved images on a Si surface at different peak fluences of 0.7, 0.8, 0.9, 1.0, and 1.7 J/cm^2^. All fluences are higher than the Si ablation threshold (0.43 J/cm^2^) [30], determined by the method introduced by Liu [31].

Figure 2 demonstrates that (1) reflectivity increases between 0 and 1 ps; (2) a region of an extremely low reflection is formed between delay time of 30 and 500 ps for the fluence between 0.9 and 1.7 J/cm^2^; and (3) after Stage 2, growing Newton rings gradually appear and expand between tens of picoseconds and one nanosecond.

**Figure 3:**
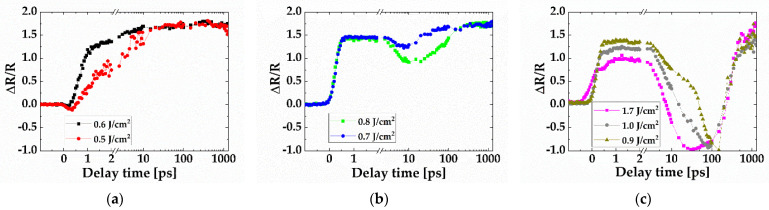
The temporal evolution of the surface reflectivity at increasing peak fluences (**a**) 0.5 to 0.6 J/cm^2^, (**b**) 0.7 to 0.8 J/cm^2^, and (**c**) 0.9 to 1.7 J/cm^2^. The Si ablation threshold is 0.43 J/cm^2^.

The time evolutions of the relative reflectivity measured at the center of the excited area at increasing peak fluences are shown in Figure 3. The reflectivity evolution strongly depends on the irradiation fluence. The measurement results are grouped into three typical cases according to the fluence values in comparison to the Si ablation threshold.

For fluences slightly above the ablation threshold (Figure 3a), a decrease in reflectivity relative to the initial value was observed at a delay time less than 0.7 ps. Afterward, the reflectivity increases and reaches an equilibrium state at about 10 ps.

For fluence values near two times the ablation threshold (Figure 3b) and less than four times the ablation threshold (Figure 3c), reflectivity increased and reached a stable state around 0.7 ps. It remained at the stable state for several picoseconds then decreased. The reflectivity at the stable state was inversely related to the fluence of the pump beam. The theoretical explanation is provided in Section 3.3.

The delay time at which the reflectivity starts to drop is related to the fluence of the pump beam. The reflectivity begins dropping from the stable state at a delay time of several picoseconds when the fluence was near two times the ablation threshold (Figure 3b). It reached a local minimum value (>1) at a time delay of approximately 10 ps. It then went back up to another stable state. The reflectivity decreases significantly when the fluence exceeds two times the ablation threshold (Figure 3c). It reached a minimum value that was lower than the initial level at a time delay ranging from 30 ps to 150 ps (30, 80, and 150 ps for 1.7, 1.0, 0.9 J/cm^2^, respectively). The decrease of the relative reflectivity not only represents the relaxation of plasma density but also indicates the change of material state. As explained by the hydrodynamic model of material ablation [10], laser excitation leads to isochoric heating of the material to an extremely hot, pressurized fluid state. Therefore, the material exhibits high absorption and low reflection, behaving like a black body.

**Figure 4:**
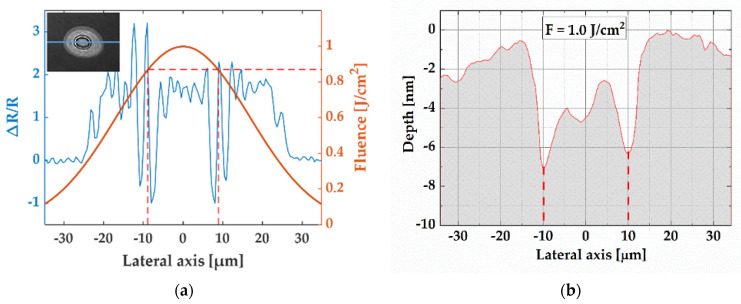
(**a**) Cross-sectional profile (blue curve) along the horizontal blue dashed line in the inset and the corresponding spatial fluence distribution (red curve). The red dashed line indicates the radius of the white rings and the corresponding fluence of 0.87 J/cm^2^. Inset: dynamic image at a delay time of 1 ns with the peak fluence of 1.0 J/cm^2^ (Figure 2 (Row 2, Column 7)). (**b**) Vertical profile of the final ablation crater measured with a ZYGO white-light-interferometer microscope. The red dashed line indicates the position corresponding to the white rings shown in Figure 4a.

Figure 4 compares the dynamic image and the final crater produced by a fluence of 1.0 J/cm^2^. Figure 4a shows the cross-sectional profile of the dynamic image at a delay time of 1.0 ns (Figure 2 (row 2, column 7)). It is overlayed with the Gaussian-shaped fluence profile of the laser beam (radius of 33.5 μm at 1/e^2^ of peak fluence) to access the material response at different local fluences after a single pulse excitation. The black ring shown in the inset in Figure 4a defines the boundary of two regimes in the dynamic image: (1) the first ablation regime corresponds to the periphery of the crater with lower local fluence; (2) the second ablation regime corresponds to the central region with higher local fluence. In the second region, the intense multiphoton ionization and avalanche ionization induced by the excitation at high fluence lead to more energy being absorbed in a very thin layer close to the surface. The material is quickly removed from the surface, developing a much shallower crater. This is the reason for a sudden decrease in depth [32]. Figure 4b shows the ablation crater profile measured by a white-light interferometer.

As shown in Figure 4a, the local fluence that induces the second ablation region was 0.87 J/cm^2^, approximately twice the Si ablation threshold (0.43 J/cm^2^). The previous investigations on different materials also identifies two distinctive regions for the laser-induced modifications, and found that the threshold for the second region was approximately twice the threshold for the first region [33,34].

**Figure 5:**
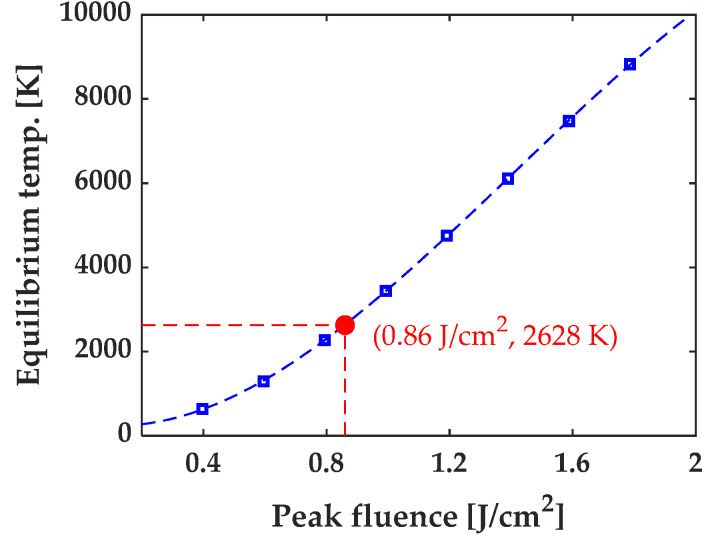
Equilibrium temperature extracted from TTM simulation results obtained at different fluences, red point indicating that boiling temperature of Si (2628 K [35]) is reached at a fluence of 0.86 J/cm^2^.

We further simulated lattice temperature at different laser fluences, using a Two-Temperature model (TTM) that will be described in the following section. Figure 5 shows that lattice temperature increased with increasing laser fluence, and silicon boiling temperature (2628 K [35]) was achieved at ~0.86 J/cm^2^. This indicates that the induction of the second ablation region is likely related to the fact that the material reaches its boiling temperature within this region.

The reflectivity evolution at the fluence (0.86 J/cm^2^) corresponding to the boiling temperature is expected to be in between what was measured at the fluence of 0.8 and 0.9 J/cm^2^, just below and above the boiling fluence. As shown in Figure 3b,c, the reflectivity at the two fluences first increases and reached a temporarily stable state with similar time scale and magnitude, then decreased with significantly different magnitude. The reflectivity decreased moderately from 1.4 to 0.875 for fluence of 0.8 J/cm^2^, whereas it decreased significantly from 1.375 to −1 for fluence of 0.9 J/cm^2^, forming a significantly darkened area that corresponds to an extremely hot fluid state. Similar decreases in reflectivity change and dark-area formation were observed for fluence above 0.9 J/cm^2^.

### 3.2. Analysis of Electron-Hole (e–h) Plasma Generation

The pump-probe images describe how the temporal evolution of the surface reflectivity of the excited sample depends on the laser fluence. The reflectivity change was directly influenced by plasma generation during the laser–material interaction. We study the temporal evolution of plasma generation in relation to laser fluence using a TTM. We further study the relationship between the reflectivity and plasma density using the Drude model. We integrate the TTM and Drude models to link the temporal evolution of the reflectivity to laser fluence.

#### 3.2.1. Two Temperature Model to Explain Plasma Generation

A TTM is employed to simulate three major phenomena in Si [30]: plasma generation, plasma temperature evolution, and lattice heating using Equations (2)–(4), respectively.

(2) $$\frac{\partial N_{e-h}}{\partial t}=G_{c}-D_{c}-\nabla \cdot \vec{J}\;$$

(3) $$C_{e-h}\frac{\partial T_{c}}{\partial t}=S-\left[\Gamma \left(T_{c}-T_{l}\right)+\nabla \cdot \bar{W}+\frac{\partial N_{e-h}}{\partial t}\left(E_{g}+3k_{b}T_{c}\right)+\frac{\partial E_{g}}{\partial t}\cdot N_{e-h}\right]\;$$

(4) $$\rho C_{l}\frac{\partial T_{l}}{\partial t}=\Gamma \left(T_{c}-T_{l}\right)+\nabla \cdot \left(\kappa_{l}\nabla T_{l}\right)\;$$

Equation (2) calculates the e–h plasma density, *N*_e–h_, which is dependent on plasma generation (*G_c_*), plasma depletion (*D_c_*), and plasma flow that is according to current density, $\vec{J}$. Equation (3) calculates the e–h plasma temperature ($T_{c}$) which is increased by energy absorption, S, and decreased by transporting thermal energy to the material lattice (with temperature, $T_{l}$). The plasma temperature is also influenced by ambipolar diffusion of electrons with a current $\bar{W}$, change in the plasma kinetic energy, and change in the material bandgap energy $E_{g}$. Here, $C_{e-h}$ is the heat capacity specific to e–h plasma and $k_{b}$ is the Boltzmann constant. Equation (4) calculates the heating of the material lattice. The temperature of the material lattice is impacted by both the plasma energy coupling and heat diffusion through the material bulk. Here, ρ is the material density, $C_{l}$ and $\kappa_{l}$ represent the specific heat capacity and thermal conductivity, respectively.

**Figure 6:**
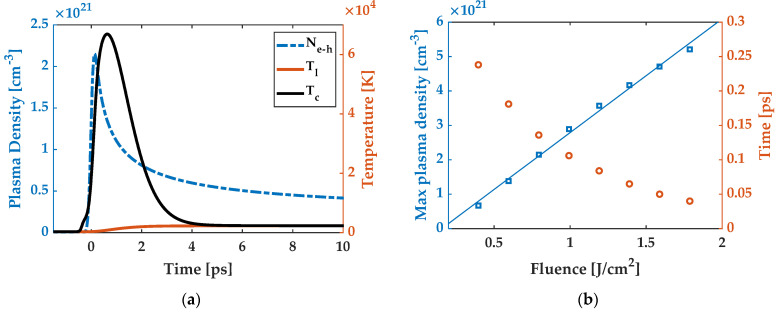
(**a**) Predicted plasma density, electron temperature, and lattice temperature with respective maxima of 2.2 × 10^21^/cm^3^, 6.7 × 10^4^ K, and 2274 K for a TTM simulation at the laser fluence of 0.8 J/cm^2^. (**b**) Maximum plasma density and time for reaching corresponding max plasma density as a function of excitation fluence (taken from TTM simulation).

Figure 6a shows an example of the TTM prediction for laser–silicon interaction at fluence of 0.8 J/cm^2^, one of the experimental values. The plasma density rises and reaches a maximum value of 2.2 × 10^21^/cm^3^ in less than 0.15 ps after the arrival of the peak of the pulse. The plasma temperature rises to a peak value of 6.7 × 10^4^ K then the heat transports to the lattices through collisional interaction. The lattice temperature equilibrates at 2274 K, approximately 4 ps after the arrival of the peak of the pulse.

A linear relationship between plasma density and laser fluence was obtained from our TTM simulation (Figure 6b), which has also been demonstrated in reference [9]. The TTM further determines that the time it takes to reach the peak plasma density, or the velocity of plasma generation, is in the sub-picosecond region. It also decreases with increasing fluence (Figure 6b). It takes only 40 fs for the plasma density to reach the maximum at fluence of 1.7 J/cm^2^, which is on the order of the temporal resolution of the pump-probe imaging. This can explain why the initial decrease in reflectivity was not observed at higher fluences (0.7–1.7 J/cm^2^), as shown in Figure 3b,c. To further link the reflectivity change with plasma density and time scale, a Drude model is introduced in the next sub-section.

#### 3.2.2. Drude Model to Explain the Reflectivity Change with Plasma Density

The reflectivity change occurs within 0.7 ps post the incidence of the laser pulse, shown in Figure 3, is attributed to the generation of e–h plasma during laser–material interaction. The dielectric constant of the excited material $\varepsilon^{*}$ in terms of free carriers can be calculated using the Drude model, as shown by Equation (5) [9,36]:

(5) $$\varepsilon^{*}=\varepsilon_{c-\mathrm{Si}}-\frac{N_{e-h}e^{2}}{\varepsilon_{0}m_{opt}^{*}m_{e}\omega^{2}}\frac{1}{1+i\frac{1}{\omega \tau_{D}}}\;$$

wherein, $\varepsilon_{c-\mathrm{Si}}$ is the dielectric constant of the unexcited material at the probe-beam wavelength, 515 nm. $\varepsilon_{0}$ is the vacuum dielectric permittivity, ω is the frequency of incident light, *m*_e_ and *e* are the electron mass and charge, respectively. $m_{opt}^{*}$ denotes the optical effective mass coefficient of the carriers, *N*_e–h_ is the density of excited e–h pairs. The Drude damping time $\tau_{D}$ represents the electron–electron collision time, which is inversely proportional to the density of the plasma generated ($N_{e-h}$), $\tau_{D}=\tau_{0}\frac{N_{cr}}{N_{e-h}}$ [23,24,26]. $\tau_{0}$ is the damping constant without laser excitation. The critical plasma density is defined as $N_{cr}$ when $Re\left(\varepsilon^{*}\right)=0$ [9]. It is 6.9 × 10^20^/cm^3^ for silicon [30]. The refractive index of e–h plasma is expressed as $n_{e-h}=\sqrt{\varepsilon^{*}}$.

The reflectivity at the air–plasma interface can be calculated using Fresnel equation (Equation (6) [37]):

(6) $$R_{p}={\left|\frac{n_{e-h}/\cos\theta_{e-h}-n_{c-\mathrm{Si}}/\cos\theta_{\mathrm{air}}}{n_{e-h}/\cos\theta_{e-h}+n_{c-\mathrm{Si}}/\cos\theta_{\mathrm{air}}}\right|}^{2}$$

wherein *θ*_air_ and *θ*_e–h_ are the incidence angle in air and refractive angle in e–h plasma layer, respectively. The complex-valued refractive index $n_{c-\mathrm{Si}}$ is defined as $\sqrt{\varepsilon_{c-\mathrm{Si}}}$, and it is 4.2410 + i0.0893 (T = 300 K) for monocrystalline Si [38].

To interpret the experimental results demonstrated in Figure 3, we further integrate the TTM and Drude models to obtain the relation between reflectivity change and interaction time (ΔRR versus t). Here, the reflectivity change with plasma density (ΔRR versus *N*_e–h_) is determined by the Drude model; the temporal evolution of plasma (*N*_e–h_ versus *t*) is predicted by the TTM.

**Figure 7:**
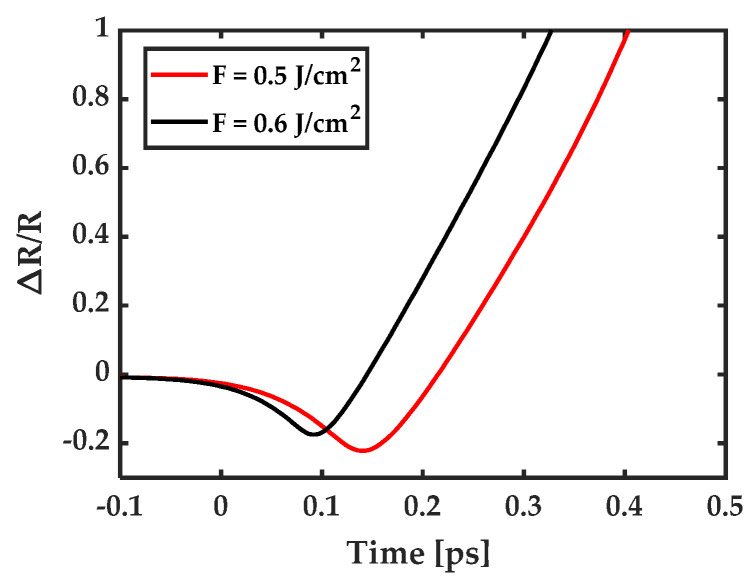
Relative reflectivity as a function of delay time. The time zero aligns with the experimental data.

Figure 7 shows the predicted reflectivity by the integrated model. It slightly decreases to a local minimum, then increases to 1 in less than 0.4 ps. The time it takes to reach the local minimum is 0.1 and 0.14 ps for fluence of 0.6 and 0.5 J/cm^2^, respectively. The decrease in plasma-induced reflectivity is smaller for the higher fluence of 0.6 J/cm^2^ compared to the lower fluence of 0.5 J/cm^2^. This is consistent with the experimental observation shown in Figure 3a, in which the initial decrease in reflectivity is also smaller for fluence of 0.6 J/cm^2^ than that for 0.5 J/cm^2^.

### 3.3. Analysis for the Saturation of Reflectivity

**Figure 8:**
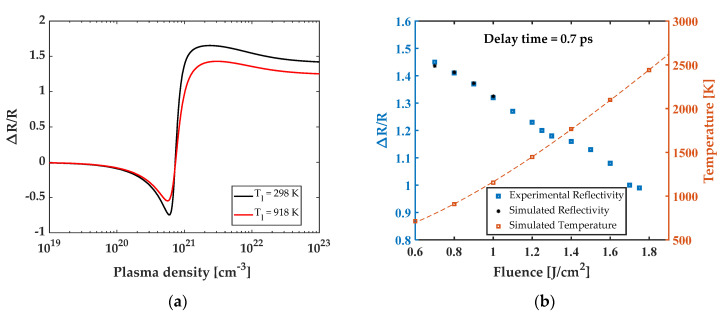
(**a**) Comparison using the refractive index at room temperature and at the predicted temperature when the laser fluence is 0.8 J/cm^2^. (**b**) Theoretical reflectivity characteristic and maximum lattice temperature as a function of fluences when taking temperature-dependent refractive index.

The impact of the temperature dependence of refractive index on the reflectivity calculation is further studied. Figure 8a shows that the reflectivity is over predicted when using the refractive-index value at room temperature, compared to using the TTM-predicted temperature for laser fluence of 0.8 J/cm^2^. It also shows that the relative reflectivity drops from 0 to −0.55 when the plasma density increases from 10^19^ to 5.6 × 10^20^/cm^3^, then increases to 1.41 when the plasma density reaches 2.2 × 10^21^/cm^3^. This model determined reflectivity agrees well with the measured value of 1.45 at 0.8 J/cm^2^ (Figure 3b).

Figure 8b shows good agreement between the experimentally measured and the simulated saturated reflectivity at 0.7 ps. The relative reflectivity decreases with increasing temperature, induced by increasing fluence. It is worth noting that reflectivity is simulated only up to fluence of 1.0 J/cm^2^, due to the data limitation of temperature-dependent refractive index [38].

## 4. Conclusions

We have studied the evolution of e–h plasma generation and ablation in Si excited by a single fs laser pulse at various fluences. The reflectivity change strongly depends on the peak fluence. Two ablation regimes have been experimentally identified, and the threshold of the second regime is theoretically determined to be 0.86 J/cm^2^ which produces the boiling temperature. We accurately predict plasma generation and lattice temperature using a TTM. We further integrate the TTM with the Drude model to provide a comprehensive understanding of the dynamic process of plasma excitation and material ablation. At the initial stage after excitation, the decrease of reflectivity at the lower fluences is observed experimentally and confirmed by the Drude model. The increasing velocity of plasma generation with increasing fluence interprets why the decrease of reflectivity is not experimentally observed. Furthermore, the observation that the saturated reflectivity decreases with increasing fluence is theoretically confirmed by integrating the TTM and Drude model and using a temperature-dependent refractive index.
